# Early combination of albumin with crystalloids administration might be beneficial for the survival of septic patients: a retrospective analysis from MIMIC-IV database

**DOI:** 10.1186/s13613-021-00830-8

**Published:** 2021-03-10

**Authors:** Shiyu Zhou, Zhenhua Zeng, Hongxia Wei, Tong Sha, Shengli An

**Affiliations:** 1 Department of Biostatistics, School of Public Health (Guangdong Provincial Key Laboratory of Tropical Disease Research, Guangdong Provincial Key Laboratory of Construction and Detection in Tissue Engineering), Southern Medical University, No. 1838 North Guangzhou Avenue, Guangzhou, 510515 People’s Republic of China; 2 Department of Critical Care Medicine, Nanfang Hospital, Southern Medical University, Guangzhou, 510515 People’s Republic of China

**Keywords:** Sepsis, Fluid therapy, Restricted mean survival time, Albumins, Mortality

## Abstract

**Background:**

Fluid therapy is a cornerstone in the treatment of sepsis**.** Recently, the guidelines have recommended the combined administration that using crystalloids plus albumin for septic patients, but the optimal timing for albumin combined is still unclear. The objective of this study was to investigate the association of timing of albumin combined with 28-day mortality in patients with sepsis.

**Methods:**

We involved septic patients from the Medical Information Mart for Intensive Care (MIMIC)-IV database, and these patients were categorized into crystalloids group (crystalloids alone) and early combination group (crystalloids combined albumin at 0–24 h). The primary outcome was 28-day mortality. We used propensity score matching (PSM) to adjust confounding and restricted mean survival time (RMST) analysis was conducted to quantify the beneficial effect on survival due to the combination group.

**Results:**

We categorized 6597 and 920 patients in the “crystalloids alone” and “early combination”, respectively. After PSM, compared to the crystalloids group, the combination group was associated with the increased survival among 28-day (increased survival: 3.39 days, 95% CI 2.53–4.25; *P* < 0.001) after ICU admission. Patients who received albumin combination at the first 24-h was associated with prolonged LOS in ICU (10.72 days vs. 8.24 days; *P* < 0.001) but lower risk of 28-day mortality (12.5% vs 16.4%, *P* = 0.003) than those received crystalloids alone.

**Conclusion:**

In septic patients, receiving albumin combined within the first 24-h after crystalloids administration was associated with an increment of survival in 28 days.

## Background

Sepsis, a life-threatening organ dysfunction resulting from infection, remains a common cause of death in critically ill patients [1]. Worldwide, sepsis affects over 30 million patients with approximately 5.3 million people dying per year [2], the prevention and treatment of sepsis have become a global health priority. Recently study reports that sepsis, in the USA, affects over 1.7 million patients and accounts for more than 50% of mortality in hospitals annually [3]. Due to the published guidelines for sepsis, the advance of machines, and antibiotic therapy, the mortality of sepsis has been decreased in these years, but still stayed at a high level [4– 8].

Fluid therapy, which is a cornerstone in the treatment of sepsis and associated with outcome in emergency department patients, is commonly used to restore and maintain patients’ tissue perfusion [9– 11]. For many years, the types of fluid administration regarding “crystalloids” and “colloids” is under the heated debate [12, 13]. Many prior studies have found that patients receiving colloids, compare to crystalloids, were not associated with decreased mortality [14, 15]. Only a few subgroup analyses showed that the colloids group may potentially reduce mortality [14, 16]. Therefore, crystalloids, as they are safe, inexpensive, and widely available, remain the first line of fluid administration in patients with sepsis [17]. Nevertheless, colloids, especially albumin, for its larger molecular weight, can stay longer in the intravascular space and expand the volume efficiently [18]. Hence, the combined administration that using crystalloids plus albumin for initial resuscitation and subsequent intravascular volume replacement has been widely used for septic patients in fluid therapy and was recommended by Surviving Sepsis Campaign Guidelines [19]. However, it remains unclear when albumin should be combined after the initiation of fluid therapy.

In this study, we sought to evaluate whether albumin combined within a specific time, compared to receiving crystalloids alone, would have a beneficial effect on survival in patients with sepsis. Additionally, we further examined the association of the different timing for albumin combined with crystalloids and increased survival. Since 2004, the guideline had recommended sets of bundles, for instance, 6-h, 3-h, and recently 1-h bundles [20– 23], highlighting that the treatment of sepsis is a “time matters” campaign. Considering the realizability in the intensive care unit, “within the first 24-h” might be an optimal time for the combination of albumin and crystalloids. We, therefore, hypothesized that combining with albumin within the first 24-h after initiation of crystalloids administration, would increase the survival time in patients with sepsis.

## Methods

### Study population

This study was a restrictive observation study from the Medical Information Mart for Intensive Care IV (MIMIC-IV version 0.4) database from 2008 to 2019 [24]. An individual who has finished the Collaborative Institutional Training Initiative examination (Certification number 35931520 for author Zhou) can access the database. This is a longitudinal, single-center database including 257,366 individuals and 196,527 adults, and 11,263 patients with sepsis (Defined by sepsis-3 criteria [1]). In our study, we extracted patients’ parameters containing age, gender, ethnic group, admission type, insurance condition, the first 24-h Sequential Organ Failure Assessment (SOFA) score, Simplified Acute Physiology Score II (SAPS) score, mean arterial blood pressure (MAP), heart rate, respiratory rate, temperature, SpO_2_, total urine output during the first 24 h after ICU admission, lactate level, the use of vasopressors, weight, mechanical ventilation, renal replacement therapy (RRT), the stage of acute kidney injury (AKI), anamnesis (myocardial infarction, cancer, renal disease, cirrhosis and diabetes) and the type and volume of their fluid administration during the whole ICU stay. Vasopressors included norepinephrine, phenylephrine, epinephrine, vasopressin, dopamine, and dobutamine. For the antibiotics, Carbapenems (meropenem), Glycopeptide (vancomycin), β-lactams (ceftriaxone, cefotaxime, and cefepime), and Aminoglycosides (gentamicin and amikacin) were extracted into our analysis. In this study, types of administration for crystalloids and albumin including normal saline and lactated Ringer’s (LR) solution, while 5% and 25% HSA for colloids. The code of data extraction is available on Github (https://github.com/MIT-LCP/mimic-iv).

Adults patients (≥ 18 years) with sepsis and complete fluid administration records were screened in the analysis. The following exclusion criteria were used: (1) patients who have not received any crystalloids administration; (2) patients who received albumin longer than 24 h after the initiation of crystalloids administration or preceded the crystalloids. For patients who had ICU admission more than once, only data of the first ICU admission of the first hospital stay were included.

### Time to combined

The timing of albumin combined was defined as the time when albumin administration minus the time of crystalloids administration. To assess the association of timing with 28-day mortality, patients were divided into the crystalloids group (no albumin combined) and early combination group (0–24 h).

### Primary and secondary outcomes

The primary outcome was 28-day mortality. Secondary outcomes included 60-day mortality, length of ICU stay (LOS ICU), length of hospital stay (LOS hospital), and the total volume of crystalloids administration during the ICU.

### Restricted mean survival time analysis

The definition of restricted mean survival time (RMST) can be described as the area of the Kaplan–Meier (KM) survival curve during a prespecified timepoint [25, 26]. The difference of RMST can be explained as the difference in areas of the KM curve for two groups, which means the reduction or increment in survival time owing to the intervention group vs. the control group during the prespecified period [27, 28].

### Statistical analysis

Baseline characteristics are presented as mean (SD) and number (percentage) for continuous and categorical variables, respectively. We used *t*-test, Chi-square (χ2) tests, or Wilcoxon rank-sum test to compare the patients’ characteristics between two groups as appropriate.

Propensity score matching (PSM) was conducted to balance the baseline characteristics between the crystalloids and the combination group. thus, we used a logistic regression model to calculate the propensity score for each patient and match 1:1 for the two groups. After PSM, standardized mean differences (SMD) were used to evaluate the balance of characteristics between the two groups. A variable can be considered as an imbalance between groups when its SMD is greater than 0.1 [29].

RMST analysis was performed to detect the association between the combination therapy and outcomes, and we compared the results of RMST analysis before and after PSM. For both groups, we reported the RMST and the difference of RMST as well as the corresponding 95% confidence intervals (95%CI) of them, during the first 28 and 60 days after ICU admission.

Moreover, we conducted an additional analysis to examine whether the increased survival days due to combination therapy varied by the timing regarding the albumin combined. Patients who have combined the albumin preceded of the crystalloids or combined greater than 24-h were categorized into the preceded combination group (< 0 h) and the late combination group (> 24 h), respectively. We further examined the association between increased survival time and different combination timing of the combination group. These additional analyses were exhibit by RMST analysis before and after PSM.

Also, we conducted a sensitivity analysis restricting the type of crystalloids and albumin to normal saline, Lactate Ringer, and 5% albumin, respectively, to evaluate the robustness of our results. We also performed subgroup analyses according to age, gender, and septic shock. Multiple imputation was used for the missing value under the assumption of missing at random [30]. All statistical analyses were performed with the R package (version 4.0.1) and SAS (version 9.3). A *p* value was taken as statistically significant at *p* < 0.05 (two-sided).

## Results

**Fig. 1:**
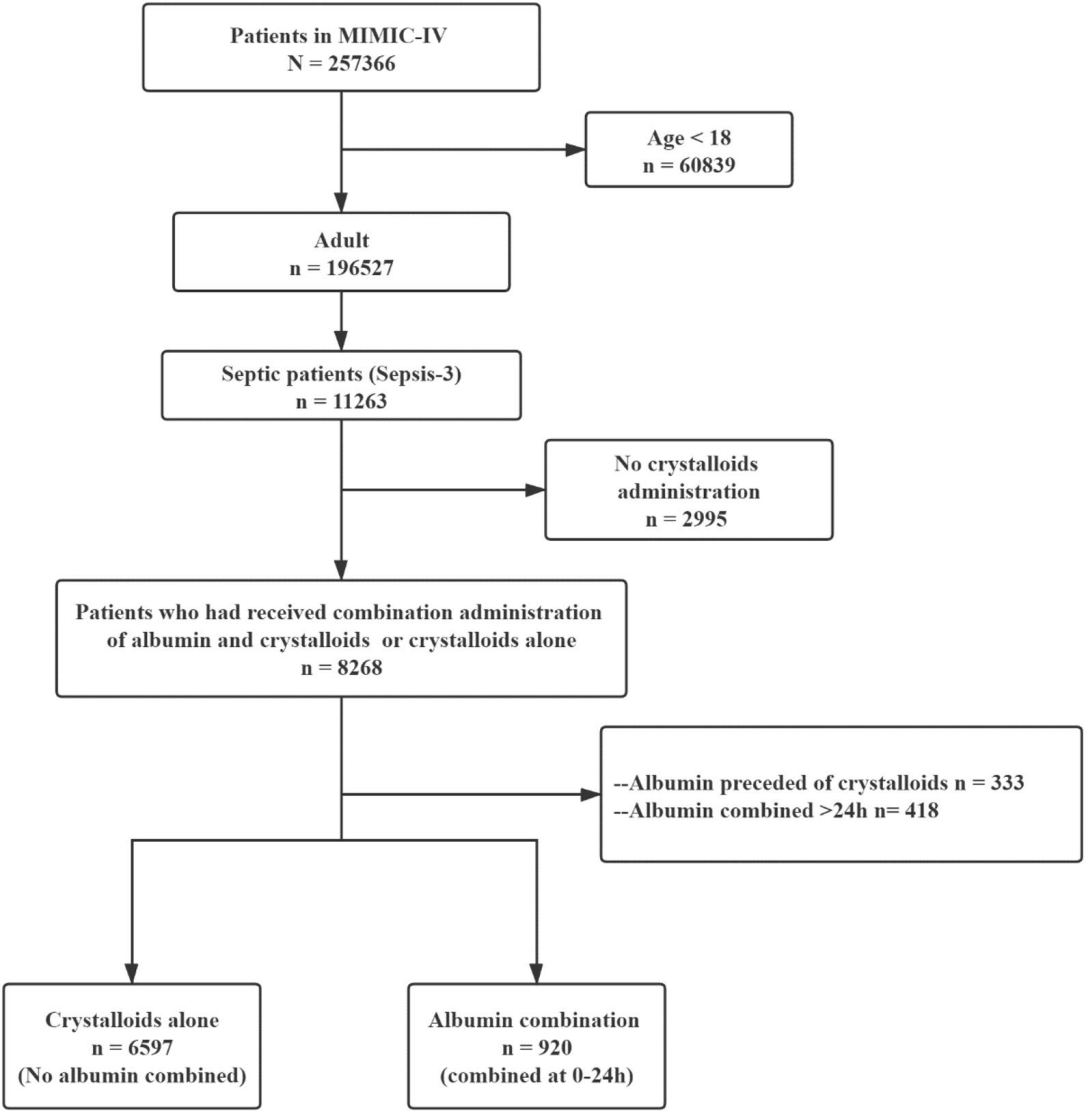
Study flowchart. *MIMIC* Medical Information Mart for Intensive Care

The MIMIC-IV database included 11,263 adult patients with sepsis, and finally, 7519 patients were included in this study (Fig. 1). Of this study, 6597 patients had used crystalloids alone as the fluid administration (group crystalloids alone), while 920 patients received albumin combined with crystalloids for the fluid administration within the first 24-h (group albumin combination).

In total, 6641 patients were included in our analysis (Table 1), the mean (SD) age was 66.75 (16.24) years, 48.57% were female, 64.82% were white person, and 5.99% had used the renal replacement therapy (RRT). Mortality was significantly lower in the combination group than in the crystalloids group (12.5% vs. 16.4%, *P* = 0.003). Similarly, respiratory rate, temperature, and urine output were significantly higher in the crystalloids group than in the combination group. Compared to the crystalloids group, patients with 3 stages of AKI, higher SAPS II score, SOFA score, and serum lactate levels were more likely to combine the albumin with crystalloids as the fluid administration within the first 24 h. A larger percentage of received mechanical ventilation (71.63% vs. 43.85% *P* < 0.001) and the use of the vasopressors (85.43% vs. 50.52% *P* < 0.001) were significantly higher among the combination group. After PSM (Additional file 1: Table S1), the SMD were all less than 0.1, indicating the baseline variables in the two groups have similar distributions.

**Table 1:** Baseline characteristics between the two groups

Variables	Total population (*n* = 7517)	Crystalloids alone (*n* = 6597)	Early combination (*n* = 920)	*P*
Age (yr)	66.75 (16.24)	67.02 (16.62)	67.11 (14.05)	0.852
Female (%)	3651 (48.57)	3253 (49.31)	398 (43.26)	< 0.001
White (%)	5359 (64.82)	4252 (64.45)	646 (70.22)	< 0.001
Heartrate (bpm)	89.98 (16.99)	89.83 (16.99)	89.39 (17.04)	0.463
MAP (mmHg)	78.14 (10.50)	78.96 (10.78)	74.10 (8.01)	< 0.001
Respiratory rate (bpm)	20.39 (4.21)	20.54 (4.21)	19.36 (3.93)	< 0.001
Temperature (°C)	36.91 (0.66)	36.93 (0.66)	36.81 (0.65)	< 0.001
SpO_2_ (%)	96.80 (2.73)	96.72 (2.78)	97.21 (2.46)	< 0.001
Urine output (L)	1.59 (1.23)	1.64 (1.27)	1.42 (1.04)	< 0.001
SAPS II score	35.45 (13.54)	34.98 (13.51)	36.88 (13.97)	< 0.001
SOFA score	4.85 (2.98)	4.64 (2.93)	5.74 (2.92)	< 0.001
Lactate (mmol/l)	2.63 (2.15)	2.50 (2.09)	2.99 (2.21)	< 0.001
Weight (kgs)	81.29 (25.66)	80.42 (25.82)	85.11 (22.70)	< 0.001
Ventilation (%)	3552 (47.25)	2893 (43.85)	659 (71.63)	< 0.001
RRT (%)	450 (5.99)	397 (6.02)	53 (5.76)	0.758
Vasopressor (%)	4119 (54.80)	3333 (50.52)	786 (85.43)	< 0.001
Admission, emergency (%)	4753 (63.23)	4363 (66.14)	390 (42.39)	< 0.001
Insurance (%)	3691 (50.73)	3269 (51.09)	422 (48.06)	0.050
AKI stage (%)				< 0.001
3	2408 (32.03)	2002 (30.35)	406 (44.13)	
2	2472 (32.89)	2127 (32.24)	345 (37.50)	
1	1008 (13.41)	902 (13.67)	106 (11.52)	
0	1629 (21.67)	1566 (23.74)	63 (6.85)	
Infection site, blood (%)	2330 (31.00)	2132 (32.32)	198 (21.52)	< 0.001
Septic shock (%)	2052 (27.30)	1828 (27.71)	224 (24.35)	0.032
Cancer (%)	1022 (13.60)	928 (14.07)	94 (10.22)	0.001
Renal disease (%)	1614 (21.47)	1426 (21.62)	188 (20.43)	0.414
Diabetes (%)	2250 (29.93)	1976 (29.95)	274 (29.78)	0.916
MI (%)	1139 (15.15)	964 (14.61)	175 (19.02)	< 0.001
Cirrhosis (%)	580 (7.72)	479 (7.26)	101 (10.98)	< 0.001
Antibiotic				
Carbapenems (%)	1095 (16.53)	938 (16.07)	157 (19.90)	0.007
Glycopeptide (%)	5785 (87.31)	5030 (86.17)	755 (95.69)	< 0.001
β-lactams (%)	1875 (28.30)	1754 (30.05)	121 (15.34)	< 0.001
Aminoglycosides (%)	147 (2.22)	124 (2.12)	23 (2.92)	0.157

*MAP* mean arterial blood pressure, *SAPS II* Simplified Acute Physiology Score II, *SOFA* sequential organ failure assessment, *RRT* renal replacement therapy, *AKI* acute kidney injury, *MI* myocardial infarction

### Timing of albumin and 28-day mortality

**Fig. 2:**
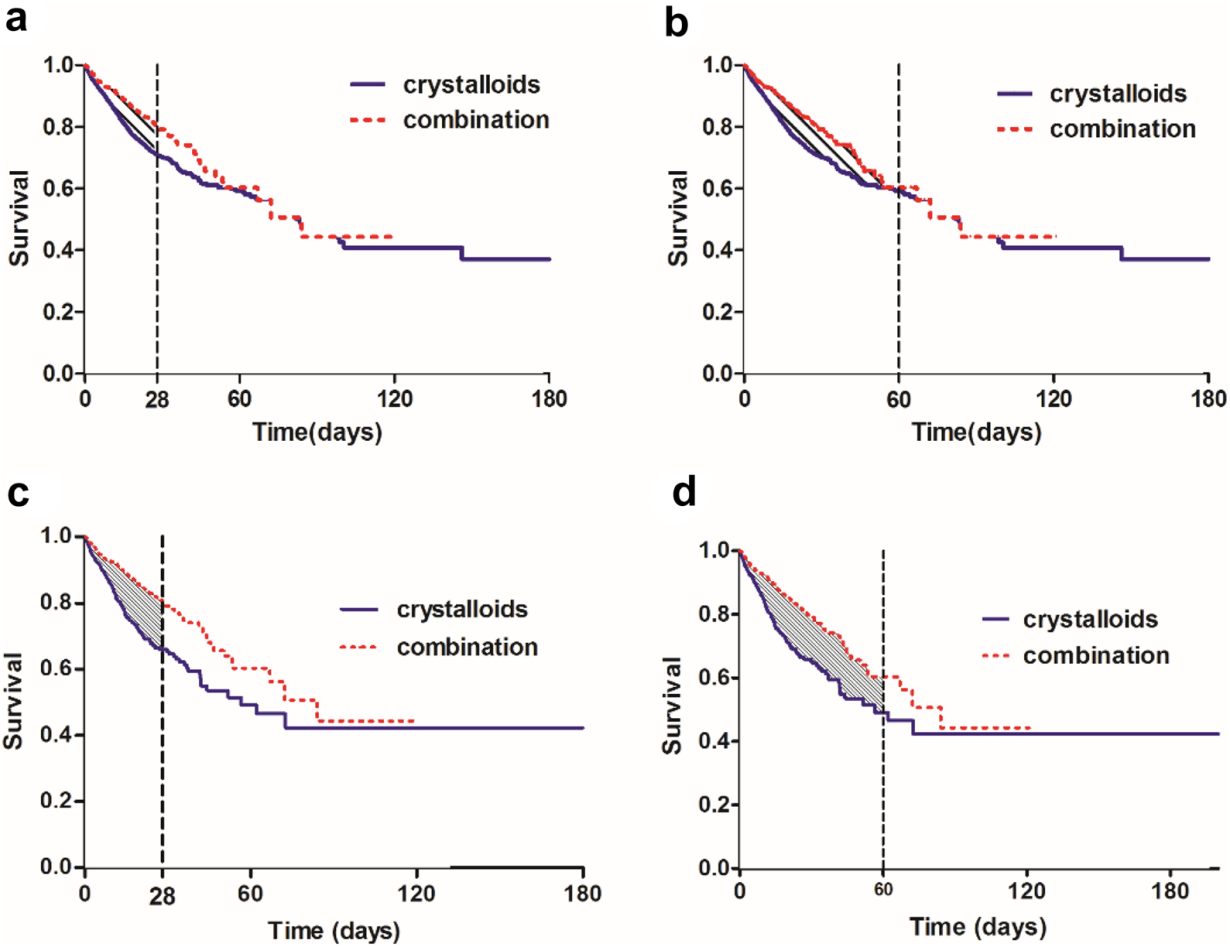
Kaplan–Meier survival curve of the two groups. Shaded regions are the difference of restricted mean survival time (RMST) for two groups among 28 (**a**, **c**), 60 (**b**, **d**) days before and after propensity score matching (1:1), means the increment of survival days due to the combination therapy among 28 and 60 days. Combination group: patients who received albumin within the first 24 h after initiation of crystalloids administration. Crystalloids group: patients who received crystalloids alone as fluid therapy

Table 2 and Fig. 2a showed that, before PSM, for the first 28 days after ICU admission, patients in the combination group were expected to live 25.10 (95% CI 24.60–25.60) days vs. 23.37 (95% CI 23.12–23.61) days in crystalloids group. Patients who received combination therapy within the first 24 h after fluid administration lived, on average, 1.73 more days (95% CI 1.17–2.29; *P* < 0.001) than the crystalloids group over 28 days. After PSM, the KM curve is shown in Fig. 2c, and as expected, the combination therapy was associated with increased 28-day survival time (3.23 days; 95% CI 2.38–4.08; *P* < 0.001). The RMST analysis indicated a beneficial effect of the combination group among 28-day follow time before and after PSM. Also, the sensitivity analysis (Additional file 1: Table S2) for the distinctive type of crystalloids and albumin revealed similar results to our main analysis (Table 2). Similar outcomes were found across subgroups regarding age (< 60 and ≥ 60 years), gender and septic shock (Additional file 1: Table S3).

**Table 2:** Primary and secondary outcomes

Outcomes	Crystalloids (*n* = 6597)		Combination (*n* = 920)		Difference (95%CI)	*P*
	Values	95%CI	Values	95%CI		
Before PSM						
RMST for 28 days (days)	23.37	23.12–23.61	25.10	24.6–25.6	1.73 (1.17–2.29)	< 0.001
RMST for 60 days (days)	46.09	45.31–46.88	50.93	49.37–52.49	4.84 (3.09–6.58)	< 0.001
LOS ICU (days)	6.65	6.47–6.83	10.37	9.65–11.09	3.72 (2.98–4.46)	< 0.001
LOS Hospital (days)	14.32	13.93–14.71	19.03	18.00–20.07	4.72 (3.61–5.82)	< 0.001
Total volume of crystalloids (L)	2.37	2.27–2.41	3.11	2.84–3.40	0.78 (0.49–1.07)	< 0.001
*After PSM (1:1)*						
RMST for 28 days (days)	22.20	21.53–22.86	25.10	24.60–25.60	3.23 (2.38–4.08)	< 0.001
RMST for 60 days (days)	42.18	40.09–44.27	50.93	49.37–52.49	9.09 (6.49–11.69)	< 0.001
LOS ICU (days)	7.73	7.25–8.21	10.37	9.65–11.08	2.64 (1.77–3.50)	< 0.001
LOS Hospital (days)	15.21	14.89–17.44	19.04	18.00–20.07	2.88 (1.24–4.52)	< 0.001
Total volume of crystalloids (L)	2.76	2.46–3.06	3.11	2.84–3.40	0.36 (-0.05–0.77)	0.085

RMST (days) means the restricted mean survival time in each group during the first 28 and 60 days after ICU admission. The difference in RMST (95%CI) was calculated with the difference of restricted mean survival time between the two groups (RMST_combination_-RMST_crystalloids_), which means the increment or reduction of survival owing to the combination therapy. *LOS ICU* Length of ICU stay, *LOS Hospital* Length of hospital stay, *PSM* propensity score matching

### Secondary outcome

In RMST analysis before PSM (Table 2), the RMST of patients among 60-day follow time was 50.93 (95%CI 49.37–52.49) days in the combination group vs. 46.09 (95%CI 45.30–46.88) days in the crystalloids group, showed that the combination group was significantly associated with improved survival time (4.84 days; 95% CI 3.09–6.58; *P* < *0.001*). However, prolonged LOS in ICU (10.37 days vs. 6.65 days; *P* < 0.001) and hospital (19.03 days vs. 14.32 days; *P* < 0.001) were significantly more likely in the early combination group vs. the crystalloids group. Also, the combination group compared to the crystalloids group was associated with the larger volume of crystalloids administration (3.11L vs. 2.37L; *P* < 0.001). After PSM, these increased survival time due to the combination therapy during 60-day follow-up time (9.09 days; 95% CI 6.49–11.69; *P* < 0.001) remained. The KM curve of two groups with the truncation of 60-day before and after PSM is reported in Fig. 2b, d, respectively. The beneficial effect of the combination group against the crystalloids group increased along with the prolonged follow-up days. Compared to patients receiving crystalloid therapy, patients in the combination group had no significant difference in the total crystalloids administration (3.11L vs. 2.76L; *P* = 0.085).

### Additional analysis

**Fig. 3:**
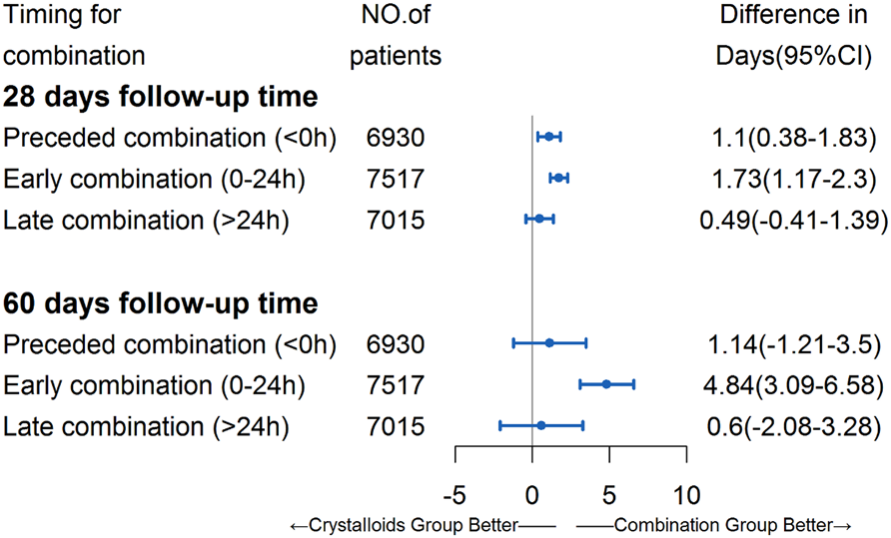
Association of increased survival and the combination group before propensity score matching. The difference in days (95%CI): The difference of restricted mean survival time (RMST) between the two groups (RMST_combination_-RMST_crystalloids_), means the increment or reduction of survival owing to the combination therapy. The preceded combination group (< 0 h) is defined as patients who received the albumin solution as the initiation of fluid administration and subsequently combined with crystalloids. The late combination group (> 24 h) is defined as patients who received the albumin greater than 24 h after crystalloids administration

In total, 1671 patients had received albumin and crystalloids during the ICU. Except for 920 patients who combined albumin at 0-24 h were included in our primary analysis, 333 (19.93%) patients were combined albumin preceded of crystalloids (< 0 h) and 418 (25.01%) patients had combined with albumin greater than 24-h after crystalloids administration (> 24 h). The comparisons of the three combination group with the crystalloids group are presented in Fig. 3. Compared to the crystalloids group, significantly increased survival among the preceded combination group was shown during the 28-day (1.10 days, 95%CI 0.38–1.83) but not found in the 60-day (1.14 days, 95%CI -1.21–3.50) follow-up. For the late combination group, there were no significant survival benefits among the first 28 and 60 days (all *P* > 0.05). After PSM, the RMST analysis (Additional file 1:Figure S1) indicated that patients who received albumin combined in three periods (< 0 h, 0–24 h, > 24 h) resulted in a significant improvement of survival than those who received crystalloids alone. But the increased survival days (survival benefits owing to combination therapy) among the preceded combination group (< 0 h, 1.98 days) and late combination group (> 24 h, 2.25 days) decreased compared to the early combination group (0-24 h, 3.23 days) during the 28 days. Similarly, the results of a 60-day follow-up were comparable with 28 days.

**Fig. 4:**
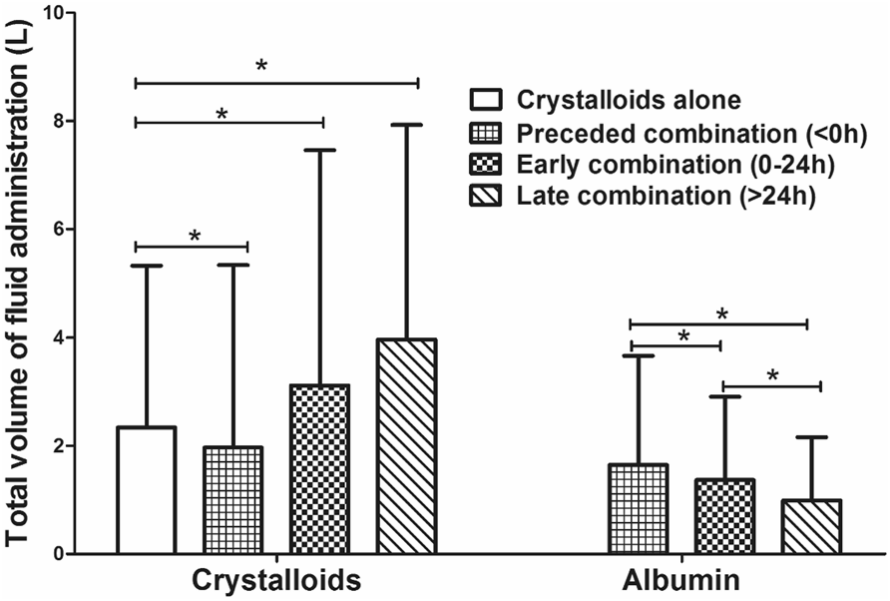
Total volume of fluid administration for each group. *: significant

**Fig. 5:**
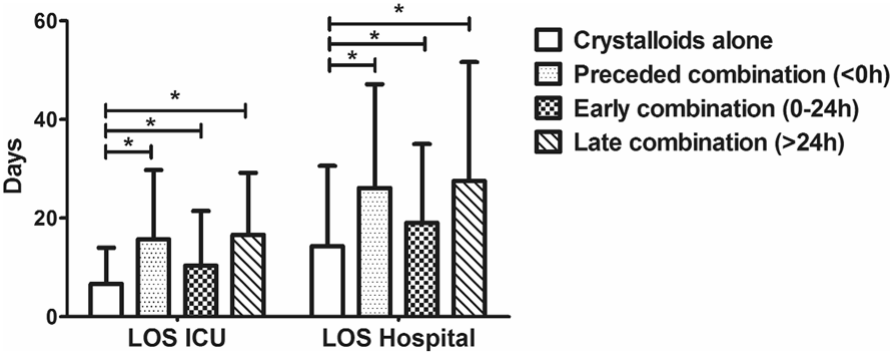
Length of stay in ICU and hospital for each group. *: significant

Considering the secondary outcomes of the three different timing groups (< 0 h, 0–24 h, > 24 h) and the crystalloids group. The preceded combination group (< 0 h) had a significantly less volume of total crystalloids administration during the ICU than the crystalloids group. Also, patients’ delay in combining albumin was associated with the larger volume of crystalloids infusion (1.9L vs. 3.1L vs. 3.9L in three timing). In contrast, patients in the late combination group (0.99L; 95%CI 0.88–1.11) were associated with less volume of albumin infusion compared with the early combination group (1.37L; 95%CI 1.27–1.47) and preceded combination group (1.65L; 95%CI 1.44–1.87), respectively (Fig. 4). The LOS of ICU and hospital in the crystalloids group were significantly shorter than the three combination groups (All *P* < 0.05). Among the three combination group, combining in 0-24 h resulted in the shortest LOS in ICU and hospital (Fig. 5, All *P* < 0.05).

## Discussion

In present study, we demonstrated the association of albumin combined within 24 h after the initiation of crystalloids administration and the increased 28-day survival time. The beneficial effect of combination therapy increased along with the prolonged follow-up time. Furthermore, the additional analysis showed that the increased survival days due to combination therapy varied by different combination timing. Compare with early (0–24 h) for albumin combined, preceded combined (< 0 h) or late combined (> 24 h) of albumin resulted in less increment in survival at three follow-up time. The benefits of survival among 28-d regarding the preceded combination group and late combination group comparing the crystalloids group revealed contradictory results before and under the PSM. Despite applying the PSM or prolonging the follow-up time, the combination therapy regarding the early albumin combined was still associated with the decreased risk of mortality and also associated with the longest increment in survival among three time of combination.

Many prior studies comparing the outcomes between the “crystalloids group” and “colloids group” in septic patients suggested that initiation administration with colloids and subsequently combined with crystalloids was associated with no difference or increased mortality. Conversely, a subgroup analysis [14] that included 1121 patients with septic shock suggested that albumin was associated with a significant survival benefit among 90 days (HR = 0.87; 95%CI 0.77–0.99). The definition of the colloids group in these studies is similar to the preceded combination group (< 0 h) mentioned in our additional analysis. Patients who accepted albumin as the initiation fluid (“ < 0 h” in Fig. 3), compared to the crystalloids group, showed a slightly decreased risk of mortality among 28 days (increment in survival: 1.10 days; 95%CI 0.38–1.83) and this beneficial association vanished among the 60 days (increment in survival: 1.14 days; 95%CI-1.21–3.50). One possible explanation is that in the few hours after the recognition of sepsis, patients were going through the profound alternations in tissue perfusion as well as the related hypovolemia and needed a timely and plentiful amount of fluid therapy [9]. Receiving albumin as the initiation of fluid therapy following crystal liquids not an advisable strategy.

Nevertheless, guidelines from SSC suggest albumin in addition to crystalloids for initial resuscitation and subsequent intravascular volume replacement in patients with sepsis and septic shock when patients require substantial amounts of crystalloids (weak recommendation, low quality of evidence) [19]. Albumin exhibits antioxidant effects and positive effects on vessel wall integrity [31]. The administration of albumin raises colloid osmotic pressure and reduces vascular permeability and adhesion of leukocytes and platelets, thus limiting edema formation [32]. Moreover, a recent pilot study demonstrated the beneficial effects on skin endothelial function after albumin administration within 24 h of septic shock [33]. The endothelial dysfunction is involved in microcirculatory blood flow impairment and associated with vasomotor tone dysregulation, activation of coagulation, and glycocalyx damage, thus, the benefit of the albumin administration within 24 h may owe to its anti-oxidant properties [33, 34]. Albumin infusion also helps maintain glomerular filtration via hemodynamic and oncotic mechanisms [35]. Albumin combined with crystalloids can balance the risk of fluid-induced adverse effects, especially in patients who need large volumes [9].

Also, Ospina-Tascon et al. [36] has reported that patients receiving 4% albumin or Ringer’s lactate solution as the fluid administration in the early phase (within 24 h) would improve microvascular perfusion but not in the late phase (more than 48 h). Additionally, a previous study evaluating the effects of repeated fluid boluses showed that only the first bolus was associated with an improvement in microvascular perfusion [37]. In the late phase of sepsis, the effect of fluid administration on tissue perfusion was limited. Similarly, Bo and colleagues [38] found that a fast rate of infusion in fluid resuscitation for septic shock patients was associated with the early shock reversal (HR = 0.78; 95% CI 0.66–0.91; *P* = 0.010) and survival gain in 28 days (HR = 0.71; 95%CI 0.60–0.85; *P* < 0.001) compare to infuse slowly. Hence, we suspected that the impact of combination therapy on survival was likewise limited. In our results, as the delay of albumin combined (> 24 h), the increment in survival they induced was decreased. Especially in 60-day follow-up, there were significantly increased survival in patients who combined albumin within 24 h after the initiation of crystalloids administration but not found in those who combined among < 0 h and > 24 h (Fig. 4). Even when applying the PSM, their increment in survival days was approximately 50% of the early combination group. It should be noted that the timeliness of albumin combined in fluid administration for sepsis is essential, a longer deferred combination indicated the less improved outcomes. In addition, we need to consider the medico-economic problem of albumin. The price of a bottle of 10 g albumin is even dozens of times the price of a bag of Ringer's lactate solution. Unnecessary use of albumin can significantly increase the patient's medical expenses. In this study, we confirmed that even within 24 h, the hospitalization cost of early albumin combined with fluid therapy is significantly increased. 

To date, there is still insufficient evidence to demonstrate the better beneficial effect of albumin than crystalloids in fluid administration for sepsis. Our study does not attempt to address this debate. Indeed, the type of fluid solution influences the outcomes in septic patients but the timing is of equal importance [36]. Hence, we magnify the importance of the timing for albumin combined in fluid administration. In our design, the crystalloids were the main component, and albumin was supplemented. On this basis, we divided the timing for albumin combined into several periods and examined whether different combined time influenced outcomes. Our findings raise the assumption that albumin combined with crystalloids might be beneficial for survival in septic patients when the time they combined is appropriate (0–24 h in our results). Additional analysis in our study compared the association of increment in survival and other timing for albumin combined and, which strengthening the robustness of our conclusion. A clear understanding of fluid administration may help decision-making in the treatment of sepsis, and lower the mortality in septic patients. Our conclusion is based on a retrospective database study. More prospective multi-center studies are needed in the future to further clarify the benefits of early albumin combined with crystal fluid rehydration in the treatment of patients with sepsis. Moreover, better monitoring of vasopressor usage would be a more natural improvement, as would a prospective randomization of individuals with sepsis and/or septic shock. In addition, the effects of the timing of albumin administration on the pulmonary edema, mechanical ventilation time, and intestinal function of septic patients are also needed to be analyzed, not just the 28-d case fatality rate, 28-d survival time and ICU hospital stay. Since albumin can maintain a higher colloidal osmotic pressure as combined fluid rehydration program, that may reduce crystalloid fluid intake and tissue edema, thus help to maintain the function of cavity organs such as lung and intestine.

The strength of this study was the RMST analysis. RMST was firstly proposed in 2010 and well established in Cardiovascular and Cancer studies as an alternative measure of the intervention effect [25, 39– 41]. At present, most randomized controlled trials and observational studies in critical care evaluated the intervention effect with the increment or reduction of risk utilizing hazard ratio (HR) and its 95% CI [39]. The interpretation of HR depended on the reference from the control group. Unlike the HR, the RMST analysis could intuitionally and validly quantify the mean survival benefits of a period for the combination therapy by directly estimating the area of survival curves [42]. Furthermore, when conducting the HR analysis (e.g., Cox proportional hazards model), the proportional hazard (PH) assumption should be met [43]. However, in practice, especially in critical care medicine, it’s hardly plausible to validate. In this condition, the intervention effect might not be significantly examined for the lacking of statistical power, while RMST analysis could neglect the PH assumption and maintain the stable estimation of the interventional effect [26].

There are several limitations to our study. Firstly, the use of propensity score matching may minimize the possible confounding factors but also substantially reduced the sample size of our study populations. Although all patients in the early combination group (< 24 h) were matched, and balance properties were satisfied (all SMD less than 0.1 in Additional file 1: Table S2), the distribution of matched dataset were less comparable to the original dataset, thus the conclusion of the results should be interpreted with caution. Secondly, although we had performed the propensity score matching to control the confounding, there might exist some residual confounders that would not be measured in this study. Thirdly, records of fluid administration for patients who had received before ICU admission were not stored in the database, which may mar our results. Fourth, in additional analysis, the definition of the combination group is determined by different timing for combination and its sample size is lower by the decrease of albumin combining periods, which may be skewed as the insufficient sample size. Fifth, because the data we based on is an observational database, the results reported in our study should be regarded only as of reference and must be further verified. Additional high-quality and larger sample size randomized trials are needed to investigate the optimal combination time for fluid administration and the optimal strategy to guide fluid therapy.

## Conclusion

In conclusion, our retrospective study confirms that different timing of albumin combined in fluid therapy may influence survival under the propensity score matching. Compared with other timing, albumin combined within 24 h after the initiation of crystalloids administration was associated with the longest increment of survival in septic patients. Considering its inherent flaw of propensity score method, further evidence of albumin combined should be verified by additional randomized trials.

## Supplementary Information

**Additional file 1: Table S1.** Baseline characteristics among two groups after propensity score matching. **Table S2.** Sensitivity analysis of restricting the type of fluid. **Table S3.** Subgroup analysis.** Figure S1. **The association of increased survival and the combination group after propensity score matching (1:1).

## Data Availability

The datasets are available in the physionet (https://physionet.org/content/mimiciv/0.4/).

## Abbreviations

MIMIC: Medical Information Mart for Intensive Care
RMST: Restricted mean survival time
PSM: Propensity score matching
SMD: Standardized mean difference
GCS: Glasgow Coma Scale
MAP: Mean arterial blood pressure
SAPS II: Simplified Acute Physiology Score II
SOFA: Sequential organ failure assessment
RRT: Renal replacement therapy
AKI: Acute kidney injury
MI: Myocardial infarction
