# Supplementary material for: Early combination of albumin with crystalloids administration might be beneficial for the survival of septic patients: a retrospective analysis from MIMIC-IV database
Source: Ann Intensive Care. 2021 Mar 10;11:42. doi: 10.1186/s13613-021-00830-8 (PMC7947075; doi:10.1186/s13613-021-00830-8)

**Additional file 1: Table S1.** Baseline characteristics among two groups after propensity score matching

| Variables | Crystalloids alone  (n = 920) | Early combination  (n = 920) | SMD |
| --- | --- | --- | --- |
| Age (yr) | 66.69 (15.80) | 67.11 (14.05) | 0.028 |
| Female(%) | 526 (57.2) | 522 (56.7) | 0.009 |
| White(%) | 289 (31.4) | 274 (29.8) | 0.035 |
| Admission, Emergency (%) | 401 (43.6) | 390 (42.4) | 0.024 |
| Insurance, Medicare (%) | 400 (43.5) | 422 (45.9) | 0.048 |
| Weight (kgs) | 84.58 (28.57) | 85.10 (22.64) | 0.020 |
| Respiratory rate (bpm) | 19.20 (3.75) | 19.36 (3.93) | 0.041 |
| Heartrate (bpm) | 89.83 (16.54) | 89.39 (17.04) | 0.026 |
| MAP (mmHg) | 74.44 (8.14) | 74.11 (8.05) | 0.041 |
| Temperature (°C) | 36.81 (0.75) | 36.79 (0.65) | 0.025 |
| SpO_2_ (%) | 97.21 (2.38) | 97.21 (2.46) | 0.001 |
| Urine output (L) | 1.44 (1.11) | 1.42 (1.04) | 0.019 |
| Lactate (mmol/l) | 2.84 (2.58) | 2.94 (2.18) | 0.041 |
| SAPS II score | 36.79 (13.42) | 36.88 (13.97) | 0.007 |
| SOFA score | 5.68 (2.88) | 5.74 (2.92) | 0.020 |
| Ventilation (%) | 661 (71.8) | 659 (71.6) | 0.005 |
| Vasopressor(%) | 787 (85.5) | 786 (85.4) | 0.003 |
| RRT (%) | 59 (6.4) | 53 (5.8) | 0.027 |
| AKI stage(%) | |  | 0.029 |
| 3 | 397 (43.2) | 406 (44.1) |  |
| 2 | 344 (37.4) | 345 (37.5) |  |
| 1 | 113 (12.3) | 106 (11.5) |  |
| 0 | 66 (7.2) | 63 (6.8) |  |
| Infection site, blood (%) | 208 (22.6) | 198 (21.5) | 0.026 |
| Antibiotic |  |  |  |
| Carbapenems (%) | 149 (16.2) | 157 (17.1) | 0.023 |
| Glycopeptide (%) | 759 (82.5) | 755 (82.1) | 0.011 |
| β-lactams (%) | 337 (36.6) | 345 (37.5) | 0.018 |
| Aminoglycosides(%) | 23 ( 2.5) | 23 ( 2.5) | <0.001 |

*MAP* mean arterial blood pressure, *SAPS II* Simplified Acute Physiology Score II, *SOFA* sequential organ failure assessment, *RRT* renal replacement therapy, *AKI* acute kidney injury, *MI* myocardial infarction

**Additional file 1: Table S2.** **Sensitivity analysis of restricting the type of fluid**

| Type of  Fluid | Outcomes | Crystalloids | | Combination | | Difference | P |
| --- | --- | --- | --- | --- | --- | --- | --- |
|  |  | Values | 95%CI | Values | 95%CI | (95%CI) |  |
| Normal  saline | Before PSM |  |  |  |  |  |  |
|  | RMST for 28-day (days) | 23.18 | 22.9-23.45 | 24.53 | 23.83-25.23 | 1.35(0.6-2.11) | <0.001 |
|  | RMST for 60-day(days) | 45.47 | 44.62-46.33 | 49.63 | 47.64-51.63 | 4.16(1.99-6.33) | <0.001 |
|  | After PSM (1:1) |  |  |  |  |  |  |
|  | RMST for 28-day (days) | 20.885 | 19.96-21.81 | 24.529 | 23.83-25.23 | 3.65(2.49-4.81) | <0.001 |
|  | RMST for 60-day(days) | 39.344 | 36.73-41.96 | 49.631 | 47.64-51.63 | 10.29(7-13.58) | <0.001 |
|  |  |  |  |  |  |  |  |
| Lactated  Ringer | Before PSM |  |  |  |  |  |  |
|  | RMST for 28-day (days) | 23.57 | 23.22-23.93 | 25.59 | 25.08-26.1 | 2.01(1.39-2.63) | <0.001 |
|  | RMST for 60-day(days) | 46.88 | 45.78-47.99 | 52.04 | 50.38-53.7 | 5.16(3.17-7.15) | <0.001 |
|  | After PSM (1:1) |  |  |  |  |  |  |
|  | RMST for 28-day (days) | 22.55 | 21.81-23.28 | 25.59 | 25.08-26.1 | 3.04(2.15-3.93) | <0.001 |
|  | RMST for 60-day(days) | 43.9 | 41.66-46.15 | 52.04 | 50.38-53.7 | 8.14(5.34-10.93) | <0.001 |
|  |  |  |  |  |  |  |  |
| 5% albumin | Before PSM |  |  |  |  |  |  |
|  | RMST for 28-day (days) | 23.37 | 23.12-23.61 | 25.06 | 24.54-25.59 | 1.69(1.11-2.28) | <0.001 |
|  | RMST for 60-day(days) | 46.09 | 45.31-46.88 | 51.02 | 49.42-52.62 | 4.93(3.14-6.71) | <0.001 |
|  | After PSM (1:1) |  |  |  |  |  |  |
|  | RMST for 28-day (days) | 22.11 | 21.42-22.81 | 25.06 | 24.54-25.58 | 2.95(2.08-3.82) | <0.001 |
|  | RMST for 60-day(days) | 42.94 | 40.87-45.02 | 51.02 | 49.42-52.62 | 8.08(5.46-10.70) | <0.001 |

RMST (days) means the restricted mean survival time in each group during the first 28 and 60 days after ICU admission. The difference in RMST (95%CI) was calculated with the difference of restricted mean survival time between the two groups (RMST_combination_-RMST_crystalloids_), which means the increment or reduction of survival owing to the combination therapy.

**Additional file 1: Table S3** Subgroup analysis

| Subgroup (N) | PSM adjustment | Difference in RMST among 28-day | | Difference in RMST among 60-day | |
| --- | --- | --- | --- | --- | --- |
|  |  | HR(95%CI) | P | HR(95%CI) | P |
| Sepsis (N=5465) |  |  |  |  |  |
|  | Before PSM | 2.1(1.55-2.66) | <0.001 | 5.79(3.86-7.71) | <0.001 |
|  | After PSM | 3.47(2.58-4.36) | <0.001 | 10.01(7.07-12.95) | <0.001 |
| Septic shock (N=2052) |  |  |  |  |  |
|  | Before PSM | 0.59(-0.8-1.97) | 0.407 | 2.17(-1.5-5.84) | 0.247 |
|  | After PSM | 2.58(0.61-4.55) | 0.01 | 7.26(2.03-12.49) | 0.007 |
| Age≥60 (N=5203) |  |  |  |  |  |
|  | Before PSM | 2.47(1.79-3.16) | <0.001 | 6.93(4.7-9.17) | <0.001 |
|  | After PSM | 3.75(2.72-4.78) | <0.001 | 9.85(6.68-13.03) | <0.001 |
| Age<60 (N=2314) |  |  |  |  |  |
|  | Before PSM | 0.22(-0.76-1.21) | 0.657 | 0.64(-2.16-3.44) | 0.652 |
|  | After PSM | 2.44(0.94-3.94) | 0.001 | 6.44(2.25-10.62) | 0.003 |
| Female (N=3561) |  |  |  |  |  |
|  | Before PSM | 1.25(0.37-2.14) | 0.006 | 2.88(0.05-5.71) | 0.046 |
|  | After PSM | 2.69(1.38-4.01) | <0.001 | 7.12(3-11.23) | 0.001 |
| Male (N=3866) |  |  |  |  |  |
|  | Before PSM | 2.09(1.37-2.81) | <0.001 | 6.31(4.13-8.48) | <0.001 |
|  | After PSM | 3.4(2.3-4.5) | <0.001 | 9.81(6.46-13.17) | <0.001 |

RMST (days) means the restricted mean survival time in each group during the first 28 and 60 days after ICU admission. The difference in RMST (95%CI) was calculated with the difference of restricted mean survival time between the two groups (RMST_combination_-RMST_crystalloids_), which means the increment or reduction of survival owing to the combination therapy.

**
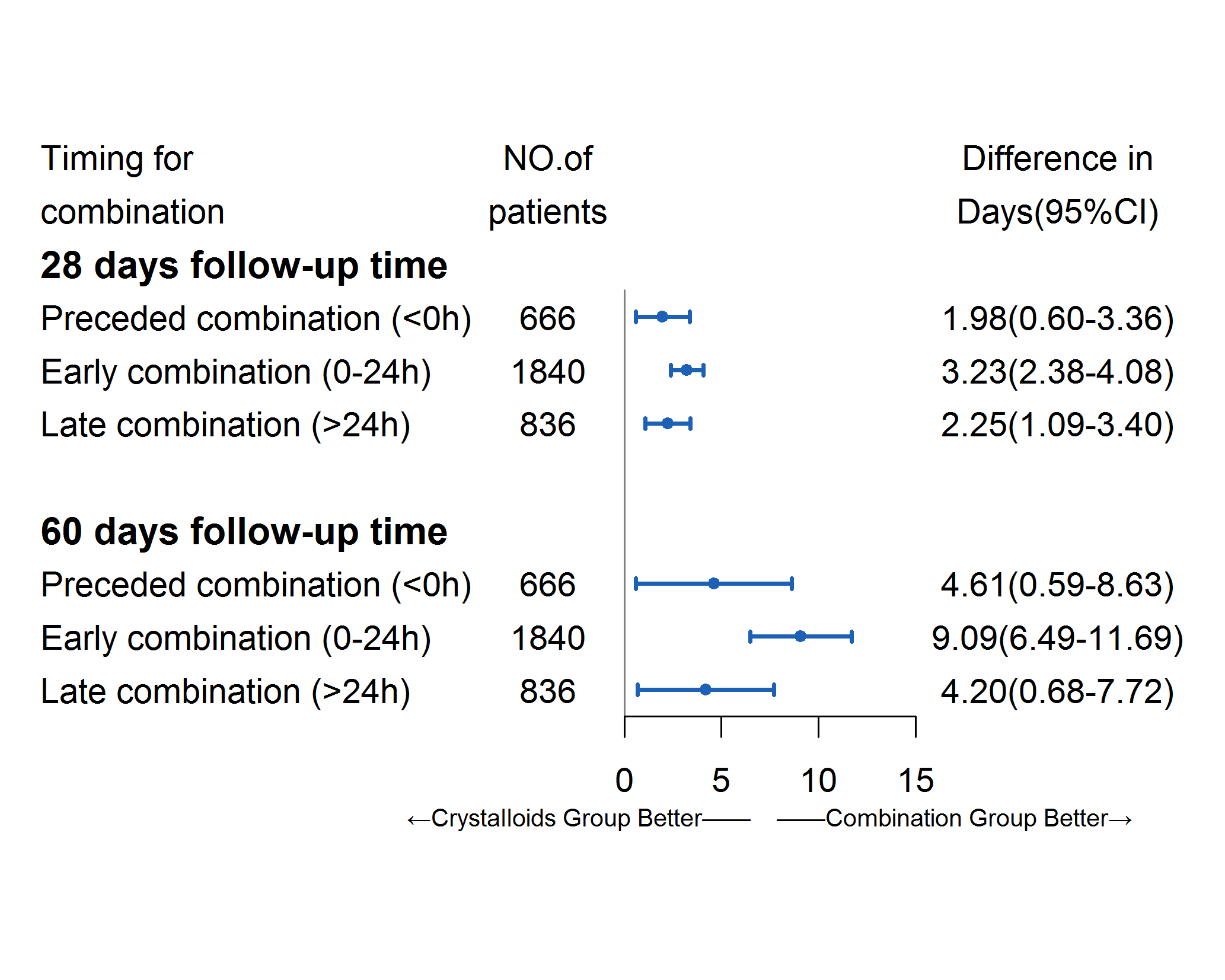
**

**Additional file 1: Figure S1.** **T****he association of increased survival and the combination group after propensity score matching (1:1)**.

The difference in days (95%CI): The difference of restricted mean survival time (RMST) between the two groups (RMST_combination_-RMST_crystalloids_), means the increment or reduction of survival owing to the combination therapy. The preceded combination group (<0h) is defined as patients who received the albumin solution as the initiation of fluid administration and subsequently combined with crystalloids; The late combination group (>24h) is defined as patients who received the albumin greater than 24 hrs after crystalloids administration.


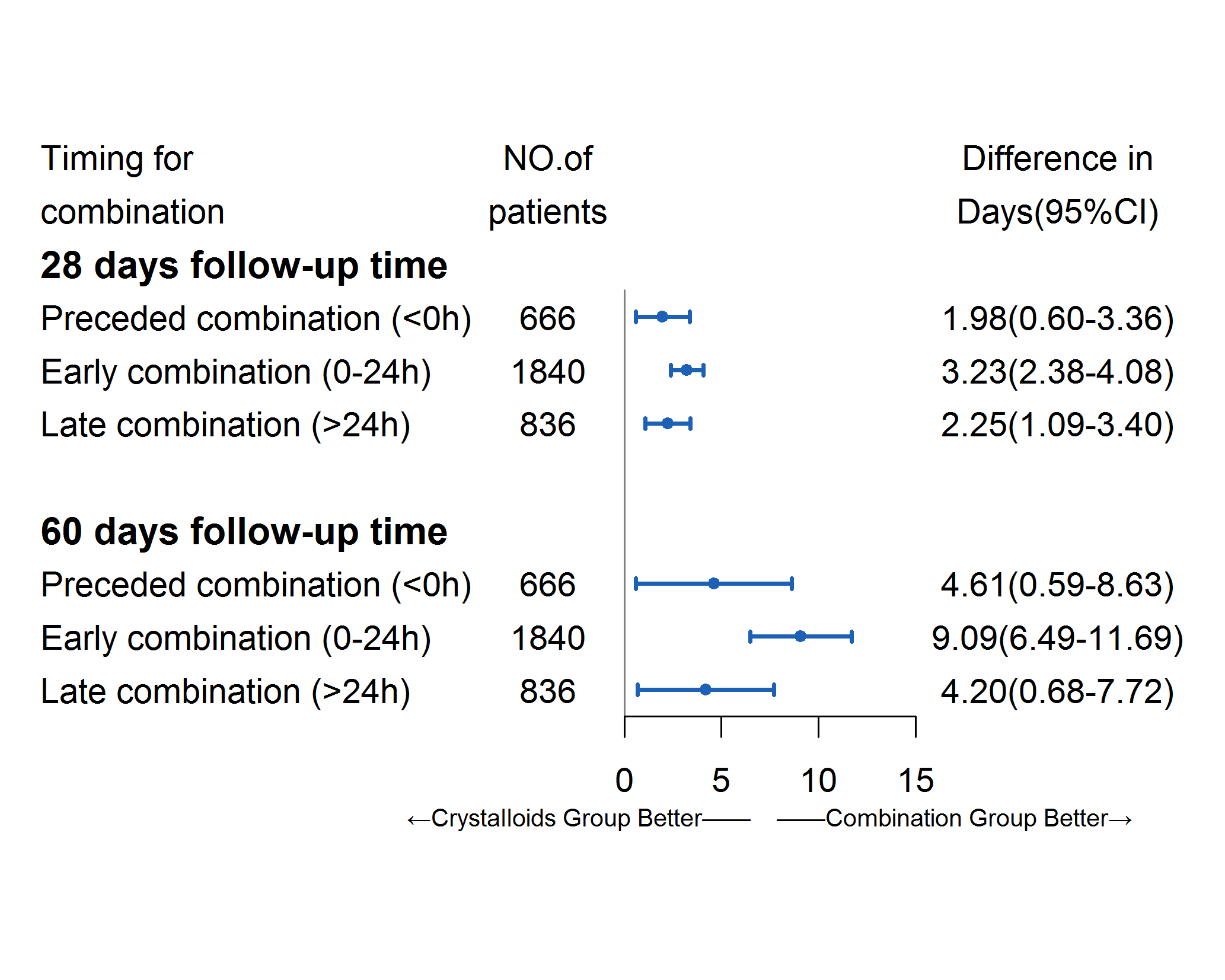

Supplement: Supplementary file 1 — Additional file 1: Table S1. Baseline characteristics among two groups after propensity score matching. Table S2. Sensitivity analysis of restricting the type of fluid. Table S3. Subgroup analysis. Figure S1. The association of increased survival and the combination group after propensity score matching (1:1). [file 13613_2021_830_MOESM1_ESM.docx]
